# Mental health of college students and their non-college-attending peers: results from a large French cross-sectional survey

**DOI:** 10.1186/s40359-016-0124-5

**Published:** 2016-04-21

**Authors:** Viviane Kovess-Masfety, Emmanuelle Leray, Laure Denis, Mathilde Husky, Isabelle Pitrou, Florence Bodeau-Livinec

**Affiliations:** EHESP French School of Public Health, Paris, France; Paris Descartes University, EA 4057 Paris, France; Institut Pasteur, Haute Autorité de Santé, Paris, France

**Keywords:** College students, Education, Health promotion, Mental health, Occupational status, Unemployment, Young adults

## Abstract

**Background:**

The great majority of mental disorders begin during adolescence or early adulthood, although they are often detected and treated later in life. To compare mental health status of college students and their non-college-attending peers whether working, attending a secondary school, or non-college-attending peers who are neither employed nor students or trainees (NENST) will allow to focus on high risk group.

**Methods:**

Data were drawn from a large cross-sectional survey conducted by phone in 2005 in four French regions in a randomly selected sample of 22,138 adults. Analyses were restricted to the college-age subsample, defined as those aged 18 to 24 (*n* = 2424). Sociodemographic, educational, and occupational status were determined. In addition, respondents were administered standardized instruments to assess mental health and well-being (CIDI-SF, SF-36, Sheehan Disability Scale, CAGE), mastery, social support, and isolation. The four occupational groups were compared. All analyses were stratified by gender.

**Results:**

Mental health disorders were more prevalent among the NENST group, with significant differences among men for anxiety disorders including phobias, post-traumatic stress disorder (PTSD) and panic disorder, impairing at least one role in their daily life. This was also true among women except for panic disorder. The NENST group also reported the lowest level of mastery and social support for both genders and the highest level of social isolation for women only. After adjustment, occupational status remained an independent correlate of PTSD (OR = 2.92 95 % CI = 1.4–6.1), agoraphobia (OR = 1.86 95 % CI 1.07–3.22) and alcohol dependence (OR = 2.1 95 % CI = 1.03–4.16).

**Conclusion:**

Compared with their peers at work or in education/training, the prevalence of certain common mental health disorders was higher among college-aged individuals in the NENST group. Efforts should be made to help young adults in the transition between school or academic contexts and joining the workforce. It is also important to help youths with psychiatric disorders find an occupational activity and provide them information, care, support and counseling, particularly in times of economic hardship. Schools and universities may be adequate institutional settings to set health promotion programs in mental health and well-being.

## Background

The great majority of mental disorders begin during adolescence or early adulthood, although they are often detected and treated later in life [1–3] due to the fact that young adults are reluctant to seek help from a mental health professional [4] or recognize that they suffer from mental health problems. [5] Reducing the burden of psychiatric disorders in young adults is critical considering their impact on academic achievement, occupational activities, social functioning and overall quality of life at a point in their life [6].

The proportion of young adults attending college varies depending on the country.

In the U.S., approximately one-half of young people aged 18 to 24 are enrolled in college [7]. In Europe, it is estimated that 24.5 % of men and 25.9 % of women aged 25 to 64 have attended college at some point. France, however, is in a unique position given the fact that tuition fees are minimal and that students who cannot afford the fee or living expenses qualify for governmental student aid which covers both, resulting in an estimating 39.1 % of students declared to receive some sort of financial allowance [8]. This unique system allows certain young adults to enter higher education though they would never have been able to in other countries in which tuition or harsh selection process is in place. Furthermore, the French system tends to delay access to employment among young adults.

Although the proportion of college students is higher in France than what is observed in other countries, non-college-attending peers also have unique circumstances. For instance, unlike many O.E.C.D. countries (Organization for Economic Co-operation and Development), where young people are entitled to welfare benefits such as jobseeker’s allowance as soon as their reach 18, youths who are neither students nor working have to wait until their 25th birthday to receive welfare benefits (Revenu de Solidarité Active). Furthermore, the unemployment rate for the age category 15–24 year-old in metropolitan France is currently running at 23.7 % (24.4 % among women and 22.9 % among men) according to the INSEE Labor Market survey^1^ as compared to 18 % for the European Union. Elevated unemployment rates among young adults in Europe and the deterioration of the labor market due to the economic crisis could have a negative effect on their mental health and well-being as they lead to social exclusion and stigmatization [9]. Together, these elements point to the importance of understanding how mental health status relates to occupational status in young adults residing in France, though this has never been specifically examined.

Comparisons of college-students and non-college-attending peers in the U.S. have shown that the prevalence of psychiatric disorders was similar in these two groups for any mood or anxiety disorder and for any alcohol use disorder when controlling for gender, race, income, region and health insurance statute, and higher in the non-college attending group for drug use disorder, nicotine dependence, bipolar disorder, conduct disorder, or personality disorder as compared to college students [10]. The latter study concluded that mental health of young people deserves more attention regardless of their occupational situation.

These findings have not been replicated in Sweden, where alcohol-related disorders are nearly four times more common among economically inactive adults aged 20–24 years than among their working or student peers, and where drug abuse is ten times more common and odds ratio (OR) for depression and self-harm were 2.5 and 3.5 respectively for this group [11]. Furthermore it has been reported that among 18–29 years old drinkers with alcohol dependence there is an increased risk of mood or anxiety disorders in non-students (OR = 4.7) as compared to students (OR = 2.4) [12].

The excess of risk for non-college attending young people has also been reported in several British surveys [13, 14] which indicated that university students who had considered dropping out of school for financial reasons had poorer mental health, lower levels of social functioning and vitality, and poorer physical health. Furthermore, in a follow-up study conducted in Japan in a female junior college, taking temporary leave or dropping out of college was associated with an unfavorable psychological state and lifestyle at the time of first enrollment [15]. Consequently, it is possible that in the NENST there are former students who were either at risk prior to entering college, or who dropped out due to their poor psychological health, thus blurring the relationship of being inactive and mental health.

While some data have been published on French college students [16, 17] or on college-age individuals [18, 19], to date, no study has focused on comparing the mental health status of college students and non-college-attending young adults who are either working or neither working nor studying, despite the fact that the latter group represented 16.2 % of this age group in France in 2010 (Eurostat),^2^ an increase from the 13.5 % estimated in 2008.

The aim of the present study is to compare the mental health status of college students and their non-college-attending peers whether working, a secondary school student, or neither in a large population-based survey using standardized assessments of psychiatric disorders. Specifically, the objectives are 1) to compare the prevalence of mental disorders and substance use problems across these groups, 2) to estimate the adjusted risk of suffering from each mental health problem associated with occupational status, and 3) to investigate gender differences in mental disorder risk by occupational status.

Our hypotheses are that in France as is the case in several other European countries where numerous social subsidies allow a large proportion of young people to attend higher education, those who are neither students nor workers constitute a minority who will have significantly more mental health problems than students or those employed. We further hypothesize that young French workers and college students will have similar proportions of mental health problems as being employed in difficult economic times reflects a certain level of adjustment. Lastly, we hypothesize that in the NENST group men have greater odds of presenting with mental health problems as compared to women.

## Methods

### Sample and procedure

Data were drawn from a large cross-sectional survey conducted in 2005 in four regions of France: Upper-Normandy, Ile-de-France, Lorraine and Rhône-Alpes. The study sample was based on a two-stage randomization method. First, households were randomly contacted: 59,836 households with landline numbers corrected for the private numbers trough the transformation of the last digit resulting in 32,397 eligible private households after exclusion of businesses and fax numbers plus those who were not reached after 15 attempts at different times during the week; second, one person was randomly selected within each household according to a method proposed by Kish [20]. Data were collected between April and June 2005 by trained interviewers using a computer-assisted telephone interviewing system (CATI). Exclusion criteria included being a non-French speaker, being a minor, being unable to answer the phone or complete the interview (the person suffered from deafness, did not answer the questions or answered inconsistently, was intoxicated, or suffered from a physical illness that prevented him or her from talking for a long period of time). After these exclusions, 26,933 persons were eligible on landline phones among these 20,077 persons participated (74.54 %). In addition to this sample, a mobile phone-only sample (response rate: 16.3 %) was collected in order to reach persons who were not equipped with a landline. Once the data were pooled, the final sample included 22,138 participants with an overall response rate of 68.72 %. Interviews lasted an average of 37 min.

Among respondents, the current study focuses on the sample of 2424 individuals who were between 18 and 24 years old with 1136 males and 1288 females. Since 26.40 % of them belong to the mobile only sample, their participation rate decreased to 54.89 %.

### Occupational status

Respondents were categorized into four mutually exclusive groups based on their current occupational status: college students (*n* = 891), non-college-attending students which included secondary school pupils and apprentices (*n* = 386), non-college-attending workers or currently employed peers (*n* = 881), and non-college-attending peers who are neither employed nor students or trainees (NENST, *n* = 266). Stay-at-home mothers (*n* = 42), persons on invalidity-related long-term leave or sick-leave (*n* = 6) and “other occupational situation” (*n* = 10) were excluded. Nineteen persons either on maternity and short-term sick-leave were considered as active and added to the group of those working or employed.

### Mental health status

Twelve-month DSM-IV axis I mental disorders were assessed with the Composite International Diagnostic Interview Short Form (CIDI-SF) [21, 22]. The full CIDI-SF was only administered to those who had endorsed gate questions on the screening portion of the instrument and assessed: anxiety disorders, major depressive episodes, and substance use disorders. The Sheehan Disability Scale (SDS) [23] was administered to assess the functional impairment associated with each disorder (SDS score ≥ 28 reflects severe impairment). In addition, the Cut-down, Annoyed, Guilt Eye-opener (CAGE) [24] scale was used to screen for possible alcohol use problems.

Respondents were also asked about suicide attempts in the previous 12 months. Psychological distressed was measured using the MH5, a subscale of the 36-item Short Form Health Survey (SF-36) [25].

### Social support, isolation, and mastery

Social support was measured with the Oslo 3-item Social Support Scale [26, 27]. This scale comprises three questions; each of them has its own answer pattern and should be used separately. One of the questions concerns the number of people close enough to rely on in case of a significant personal problem. It has been treated as a continuous variable.

The Health Canada Social Isolation Scale was used to characterize social isolation [28, 29]. This scale has four questions with a yes or no answer pattern. For analysis, answering positively to any of the four items was considered to reflect social isolation.

Finally, mastery was assessed using the sense of mastery scale [28]. This instrument has seven questions. Possible responses range from “0, totally agree” to “3, totally disagree”. The sum of the seven responses is computed and ranges from 0 to 21. A higher score corresponds to a higher level of mastery. For analysis, mastery was used as a continuous variable.

### Data analysis

Data were weighted using a Raking Adjusted Statistics (RAS)-type method taking into account gender/Age/Head of family’s occupation and socio-occupational category/Type of City/County. All analyses were run using Stata 13 software (Stata Corp Station, TX, USA) and significance threshold was set at *p* = 0.05. Chi square tests were performed to compare occupational status groups in the overall sample, among men, and among women; anovas were performed for continuous data. In addition, a series of logistic regressions were performed predicting each disorder and controlling for all other variables presented in each table.

## Results

### Sample characteristics

The proportion of females was significantly greater (*p* < .001) in the college student category (40.99 % and 31.95 %, respectively) while there were more males than females (*p* < .001) in the Worker category: (41.29 and 31.99 %, respectively) (Table 1). The youngest were the non-college-attending students followed by college students, the NENST, and the workers. Living with a partner was more frequent among the workers and the NENST as compared to college students or non-college-attending students, workers had higher income that the remaining categories.

**Table 1:** Demographic characteristics by occupational status

		Workers (*n* = 881)	NENST (*n* = 266)	College students (*n* = 891)	Non-college-attending students (*n* = 386)	p
		%	%	%	%	
Gender	Men %	53.23	46.24	40.74	46.89	**0.001**
	Women %	46.76	53.76	59.26	53.11	
Age (mean)		22.09	21.47	20.83	19.23	**0.001**
Living with partner		30.87	19.92	8.81	8.08	**0.001**
Income per person per month < = 1000 €		47.04	79.50	70.19	81.79	**0.001**
Educational attainment %	None	6.36	13.53	0.00	12.18	**0.001**
	Below secondary	38.18	43.98	0.00	87.82	
	Secondary completed	27.73	27.44	62.40	0.00	
	Bachelor	17.39	9.02	18.74	0.00	
	Masters and above	10.34	6.01	18.85	0.00	
Location	Rural	20.88	19.92	16.72	18.65	0.156
	Urban	79.11	80.07	83.28	81.35	

*Notes*: *NENST* neither employed nor students or trainees. Percentages are derived from cross-tabulations and chi-square tests

Bold means p above 0.05

Compared to males, females were more likely to live with a partner (23.06 % vs. 11.80 %, (*p* < .001), to belong to a household with less than 1000 euros per person and per month (67.66 % vs. 60.15 %, *p* < .001). There were no gender differences with respect to age, but there were differences across occupational groups.

### Prevalence of mental disorders and social support by occupational status and by gender

Depression, anxiety disorders, 12 month suicide attempts (5.75 vs. 2.82, *p* < .001), and elevated psychological distress were more frequent in women than in men. Inversely, alcohol (8.99 versus 2.41 %) and drug problems (16.08 % versus 6.07 %) were more prevalent among men than women (*p* < .001) (Table 2).

**Table 2:** Prevalence rates (%) of mental health disorders, isolation, and social support by gender and occupational status

	Men *n* = 1136						Women *n* = 1288						Total *n* = 2424					
	Total Men	Workers	NENST	College students	Non-college-attending students	p Men	Total Women	Workers	NENST	College students	Non-college-attending students	p Women	Workers	NENST	College students	Non-college-attending students	p Total	p Gender
Psychological distress	8.19	8.10	8.13	7.99	8.84	0.988	17.24	15.53	20.98	16.67	19.51	0.376	11.58	15.04	13.13	14.51	0.342	0.001
Major depressive disorder	7.66	8.32	10.57	6.61	6.08	0.347	11.88	12.86	12.59	9.85	14.63	0.260	10.44	11.65	8.53	10.62	0.346	0.001
PTSD	2.11	1.28	6.50	1.93	1.66	**0.004**	5.67	5.34	11.19	3.79	7.32	**0.004**	3.18	9.02	3.03	4.66	**0.001**	**0.001**
Alcohol problem	8.99	8.10	12.20	9.14	8.84	0.571	2.41	1.94	4.90	1.89	2.93	0.174	5.22	8.27	4.83	5.70	0.182	0.001
Drug problem	16.08	15.42	18.70	16.07	16.02	0.855	6.06	6.31	5.59	5.69	6.86	0.929	11.15	11.65	9.91	11.17	0.778	0.001
Suicide attempt	0.35	0.21	1.63	0.28	0.00	0.840	1.47	0.97	2.80	1.33	1.95	0.417	0.57	2.26	0.90	1.04	0.100	0.004
Specific phobia	5.63	7.04	9.76	3.03	4.42	**0.013**	12.38	11.17	16.08	11.20	15.35	0.196	8.97	13.16	7.87	10.18	0.061	0.001
Agoraphobia	4.14	4.05	9.76	3.31	2.21	**0.007**	12.34	10.19	16.08	11.17	17.07	**0.037**	6.92	13.16	7.97	10.10	**0.008**	**0.001**
Panic disorder	4.84	3.41	3.25	7.44	4.42	**0.043** ^a^	9.70	10.19	8.39	9.85	9.27	0.930	6.58	6.02	8.87	6.99	0.215	0.001
Social phobia	4.93	4.69	8.13	3.31	6.63	0.116	8.25	7.30	10.49	7.77	9.85	0.514	5.91	9.40	5.95	8.33	0.092	0.001
One anxiety disorder	15.75	15.48	26.02	11.57	17.88	**0.002**	30.48	27.70	35.66	28.49	37.69	**0.027**	21.19	31.20	21.56	28.31	**0.001**	**0.001**
Any disorder and impairment in each role	3.92	3.07	6.49	3.09	6.25	0.231	4.13	3.57	10.75	3.60	1.75	**0.006**	3.30	8.82	3.39	4.13	**0.009**	**0.834**
Any disorder and impairment in one role	12.86	11.48	20.88	10.36	16.08	**0.033**	19.42	17.43	26.55	16.14	27.27	**0.004**	14.33	24.02	13.81	21.89	**0.001**	**0.001**
Mastery	16.20	16.31	15.70	16.48	15.72	0.057	16.05	15.22	16.35	15.73	16.23	0.008	16.23	15.44	16.40	15.72	**0.001**	**0.540**
Isolation	22.96	20.82	31.68	23.69	20.83	0.125	10.41	8.67	15.20	8.54	15.08	0.018	14.91	22.57	14.48	17.65	**0.021**	**0.001**
Social support	3.32	3.22	3.04	3.29	3.01	0.001	3.15	3.04	3.29	3.01	3.15	0.001	3.15	3.05	3.38	3.15	**0.001**	**0.001**

*Note*: *NENST* neither employed nor students or trainees. Percentages are derived from cross-tabulations and chi-square tests

^a^NS after Bonferroni correction

Bold means p above 0.05

Overall, important differences in the prevalence of anxiety disorders and disorders associated with medium or high impairment level were observed across occupational status. In particular, any anxiety disorder, PTSD, and agoraphobia were more frequent among NENSTs. However, no significant differences were found with regard to psychological distress, major depression, alcohol or drug problems, panic disorder, phobias, and 12 month suicide attempts.

Occupational group differences varied as a function of gender. Specifically, among males, there were differences in the prevalence of specific phobia and panic disorder, while among women, there were differences in the prevalence of any disorder associated with severe role impairment.

Men appeared to be more isolated than women with 22.97 % of young men answering negatively to least at one question of the isolation scale as compared to 10.41 % of women (*p* < .001). However, men declared slightly more people they could rely on in case of a crisis as compared to women (3.32 vs. 3.14, *p* < .001). There were no gender differences with regard to mastery.

Isolation varied greatly across occupational status: 22.57 % of NENST persons answered positively at one of the isolation indicator as compared to 14.91 % of workers, 14.48 % of college students, and 17.65 % of non-college-attending students (*p* < .001). This difference persisted when controlling for gender (*p* = 0.003). The difference was mainly due to the question “could you rely on someone in case of a crisis”: 5.75 % of the NENSTs answered negatively as compared as 2.39 % of the workers, 1.91 % of college students, and 2.78 % of non-college-attending students (*p* = 0.017).

In addition, one question of the Oslo social network indicator varied by occupational status, that is the number of people close enough one can rely on in case of a significant personal problem. NENSTs’ poorer social network was then confirmed and persisted when controlling for gender. The sense of mastery of the NENST group was also lower than what was observed in the other groups.

### Predictors of mental disorders

When controlling for all other factors presented in the table, NENSTs had greater odds of PTSD (OR = 2.92 [1.40–6.07]) and agoraphobia (OR = 1.86 [1.07–3.22]) as compared to workers. College students, on the other hand had higher odds of panic disorder (OR = 2.17 [1.32–3.56]) (Tables 3 and 4). The NENSTs had higher odds of alcohol problems (OR = 2.06 [1.02–4.1]). The odds of substance-related problems were higher among those with higher incomes, as well as those not living with a partner. Those with higher levels of mastery were found to have lower odds of anxiety disorders and substance use problems. The odds of any diagnosis with severe impairment in most daily life roles were significantly higher amongst the NENST group (OR = 3.08 [1.28–7.43]) and those with social isolation (OR = 4.44 [2.25–8.76]); and significantly lower for those with higher income (OR = 0.40 [0.18–0.90]) and mastery (OR = 0.83 [0.76–0.90]).

**Table 3:** Predictors of anxiety diagnoses

*N* = 1837		PTSD				Agoraphobia				Panic disorder			
		OR	95 % CI		*P* > t	OR	95 % CI		*P* > t	OR	95 % CI		*P* > t
Women/Men		**2.84**	1.62	4.97	**0.001**	**3.04**	1.99	4.64	**0.001**	**1.48**	0.95	2.29	0.081
Occupational status (Workers as reference)	NENST	**2.92**	1.40	6.08	**0.004**	**1.86**	1.07	3.22	**0.027**	**1.17**	0.61	2.25	0.631
	Non-college-attending students	**1.33**	0.59	3.01	0.487	**1.26**	0.70	2.28	0.445	**1.81**	0.93	3.52	0.081
	College students	**1.00**	0.49	2.03	0.996	**0.97**	0.60	1.58	0.907	**2.17**	1.32	3.56	**0.002**
Age		**0.96**	0.83	1.10	0.537	**0.99**	0.89	1.09	0.783	**1.11**	1.00	1.24	0.056
Living with partner (Yes/No)		**0.98**	0.52	1.86	0.962	**1.05**	0.65	1.68	0.846	**1.28**	0.79	2.08	0.317
High/Low income		**0.86**	0.49	1.51	0.606	**0.68**	0.46	1.01	0.058	**1.17**	0.78	1.76	0.436
Mastery		**0.88**	0.82	0.93	**0.001**	**0.92**	0.88	0.96	**0.001**	**0.92**	0.88	0.97	**0.002**
Isolation (Yes/No)		**1.64**	0.89	3.02	0.110	**1.13**	0.67	1.89	0.645	**1.15**	0.70	1.89	0.586

*Note*: *OR* Odds ratios adjusting for all other variables present in the table

Bold means p above 0.05

**Table 4:** Predictors of substance problems

		Alcohol problem				Drug problem				Any disorder			
		OR	95 % CI		*P* > t	OR	95 % CI		*P* > t	OR	95 % CI		*P* > t
Women/Men		**0.22**	0.14	0.35	**0.001**	**0.33**	0.24	0.47	**0.001**	**1.67**	0.86	3.22	0.129
Occupational status (Workers as reference)	NENST	**2.07**	1.03	4.16	**0.042** ^a^	**1.05**	0.62	1.79	0.842	**3.08**	1.28	7.43	**0.012**
	Non-college-attending students	**1.66**	0.74	3.72	0.220	**0.98**	0.59	1.65	0.949	**1.03**	0.34	3.07	0.964
	College students	**1.23**	0.70	2.18	0.471	**0.91**	0.62	1.34	0.628	**1.49**	0.67	3.28	0.325
Age		**1.12**	0.98	1.28	0.102	**1.02**	0.93	1.11	0.738	**0.99**	0.81	1.21	0.933
Living with partner (Yes/No)		**0.46**	0.23	0.93	**0.032**	**0.50**	0.29	0.85	**0.010**	**1.81**	0.79	4.12	0.158
High/Low income		**1.71**	1.07	2.72	**0.024**	**1.53**	1.11	2.13	**0.011**	**0.40**	0.18	0.90	**0.027**
Mastery		**0.93**	0.88	0.98	**0.009**	**0.92**	0.88	0.95	**0.001**	**0.83**	0.76	0.90	**0.001**
Isolation (Yes/No)		**1.11**	0.67	1.84	0.676	**1.42**	0.97	2.07	0.071	**4.44**	2.25	8.76	**0.001**

*Note*: *OR* Odds ratios adjusting for all other variables present in the table

^a^NS after Bonferroni correction

Bold means p above 0.05

## Discussion

To our knowledge, the present study is the first to examine the associations between an extensive panel of mental disorders and occupational status among young adults aged 18 to 24, and to do so in a large randomly selected sample in France.

The results highlight the need to further investigate the mental health of young adults in this age group, as the prevalence of 12 month major depressive episode was 8 and 12 % for males and females respectively, and CAGE scores greater than two for 12 and 4 %, respectively. The significant differences we found between male and female respondents in our study are consistent with previous reports in the literature [30]. Similar observations were reported in Australia [31], where 11.1 % of youths aged 16–24 years have an alcohol-related disorder (8.3 % abuse and 2.7 % dependence), with males (13.1 %) more severely affected than females (8.9 %).

Our results regarding college students are in line with previous reports on French student populations [16, 17] which have found that the 12 month prevalence was 15.7 % for anxiety disorders, 8.9 % for depression, and 8.1 % for substance abuse. These results are also similar to what has been reported in college students based on the NESARC data, suggesting that 7.0 % of college students suffered from major depression, 11.9 % from any anxiety disorder, and 5.1 % from any substance use disorder in the previous 12 months [10]. Importantly, the groups had similar levels of psychological distress and the prevalence of major depression, alcohol problems, suicide attempts, panic disorder and social phobia, which is similar to what has been reported in the U.S. between college students and non-college-attending peers in adjusted models [10]. Prior studies conducted in specific student population such as medical students reported higher levels of general psychological distress and higher prevalence of depression and anxiety among U.S. and Canada as compared to peers in the general population [16, 32], although medical students may not be representative of the college student population.

Adjusted models, however, highlighted a twofold increase in the risk of alcohol use problems, when controlling for other factors was associated with being neither a student nor employed. Exposure to unemployment has been found to be significantly associated with substance abuse and criminal behavior, even after controlling for pre-existing personal and family factors [33, 34]. In France, intense use of both legal and illegal substances is found to be associated with school interruptions or dropout, and exclusion from the labor market [35], which is also the case in Sweden, where alcohol-related disorders are nearly four times more common among economically inactive adults aged 20–24 years than among their working or student peers and drug abuse is ten times more common. Adjusted models also revealed higher odds of PTSD and agoraphobia among the NENST group while in the Swedish study odds ratio for depression and self-harm were 2.5 and 3.5 respectively for this group [11].

Important differences in mastery, social support and isolation were observed as a function of occupational status. Again, those neither students nor employed displayed the less favorable circumstances. Not having a job or activity may have consequences on young people’s mental health more by reducing their social environment and network than by reducing their actual income. Further, the prevalence of social isolation was significantly higher among the male NENSTs, but not among females, suggesting that work may be a more important factor for social integration for men than it is for women. Moreover, NENSTs may develop a negative self-image and a lower self-esteem than those who attend college or are gainfully employed. Mastery and self-esteem are critical protective factors for the mental health of young adults [36]. Young people who are out of work report reduced quality of life, and quality of life is linked not only to good health but also to self-esteem, satisfaction with free time and decision latitude. For this reason, effort should aim at empowering unemployed young adults by identifying their concerns and resources [37, 38].

From a public health perspective, genuine efforts should be made to help young adults in their transition from school to the labor market. It may be important to help young people with psychological problems or psychiatric disorders finding an occupation, as unemployment is associated with lifetime disorders [39, 40]. Our results underscore the need to pay particular attention to young unemployed adults aged 18–24 years, particular in times of economic recession. In parallel, schools, universities and other educational settings can provide institutional environments for health promotion and information on mental health. For instance, in Canada, a school intervention titled The Guide and implemented by regular teachers had positive results on students’ knowledge and attitudes towards mental health [41].

The cross-sectional nature of the present study does not allow us to draw conclusions on the direction of the observed associations. However, we hypothesize that while some mental health problems are exacerbated, or even triggered by being unemployed, others may find themselves unemployed because they have always been more psychologically fragile and therefore experienced greater difficulties and adjustment problems. In a large prospective study including 5115 young adults aged 18–30 years, results showed that depressive disorders were associated with subsequent unemployment or loss of family income [42]. Similarly, a chaotic or curtailed education can be the consequence of a psychiatric disorder [43]. Psychological distress is known to be negatively associated with academic achievement which in turn has an impact on job prospects in adulthood [44]. Regardless of which came first, young people who are neither students nor employed deserve attention as a group. Future studies are needed to further investigate the circumstances of this high risk group.

Several limitations should be taken into account when interpreting the results. First, the present study was cross-sectional, precluding us from drawing conclusions as to the direction of the relationship between mental health problems and occupational status. Second, though the survey assessed the most common axis I disorders, it did not assess bipolar disorder, psychotic disorders, attention deficit/hyperactivity disorder, personality disorders and autism spectrum disorders. Third, personal and family history of mental disorders and stressful life events such as childhood abuse or neglect were not examined. Finally, the data presented here were collected in 2005. It may be important to replicate these findings in more recent data.

## Conclusion

To conclude, the present findings show that the prevalence of several common mental health disorders was higher among young adults who were not attending college and unemployed, independently from other risk factors as compared to their employed or attending college or secondary school or training. Efforts should be made to help youth adults in their transition from school to the labor market, and in times of economic recession it may be important to help young adults who suffer from psychological distress and/or psychiatric disorders secure employment and provide them with information, care, and counseling if needed.

## Ethics and consent

The study protocol was approved by the French regulation authority for questionnaire-based non-invasive medical research (“Commission Nationale de l’Informatique et des Libertés”; CNIL). All participants were given a detailed description of the study and all provided informed consent.

## Consent to publish

Not applicable.

## Data availability

The data are not made available as additional statistical analyses are currently being conducted for publication.

## Abbreviations

CAGE: cut-down, annoyed, guilt eye-opener
CATI: computer-assisted telephone interviewing
CI: confidence interval
CIDI-SF: composite international diagnostic interview short form
CNIL: Commission Nationale de l’Informatique et des Libertés
NENST: neither employed nor students or trainees
NESARC: National Epidemiologic Survey on Alcohol and Related Conditions
OECD: Organization for Economic Co-operation and Development
OR: odds ratio
PTSD: posttraumatic stress disorder
SDS: sheehan disability scale

## Footnotes

1 (http://www.insee.fr/en/themes/info-rapide.asp?id=14).

2 http://ec.europa.eu/eurostat/fr/data/database
